# Measuring and Understanding Food Insecurity in Australia: A Systematic Review

**DOI:** 10.3390/ijerph16030476

**Published:** 2019-02-06

**Authors:** Fiona H. McKay, Bronte C. Haines, Matthew Dunn

**Affiliations:** School of Health and Social Development, Faculty of Health, Deakin University, Geelong, Waterfront Campus, Geelong, Victoria 3220, Australia; bronte.haines@deakin.edu.au (B.C.H.); m.dunn@deakin.edu.au (M.D.)

**Keywords:** food security, food insecurity, Australia, measurement

## Abstract

The number of Australians seeking food aid has increased in recent years; however, the current variability in the measurement of food insecurity means that the prevalence and severity of food insecurity in Australia is likely underreported. This is compounded by infrequent national health surveys that measure food insecurity, resulting in outdated population-level food insecurity data. This review sought to investigate the breadth of food insecurity research conducted in Australia to evaluate how this construct is being measured. A systematic review was conducted to collate the available Australian research. Fifty-seven publications were reviewed. Twenty-two used a single-item measure to examine food security status; 11 used the United States Department of Agriculture (USDA) Household Food Security Survey Module (HFSSM); two used the Radimer/Cornell instrument; one used the Household Food and Nutrition Security Survey (HFNSS); while the remainder used a less rigorous or unidentified method. A wide range in prevalence and severity of food insecurity in the community was reported; food insecurity ranged from 2% to 90%, depending on the measurement tool and population under investigation. Based on the findings of this review, the authors suggest that there needs to be greater consistency in measuring food insecurity, and that work is needed to create a measure of food insecurity tailored for the Australian context. Such a tool will allow researchers to gain a clear understanding of the prevalence of food insecurity in Australia to create better policy and practice responses.

## 1. Introduction

In Australia, like other developed nations, some populations are more vulnerable to, and experience greater, food insecurity [1,2]. Food security refers to a situation when “all people, at all times, have physical, social, and economic access to sufficient, safe and nutritious food that meet their dietary needs and food preferences for an active and healthy life” [3]. This definition encompasses four hierarchical dimensions which are integral to achieve food security [4]. “Availability” refers to a reliable and consistent source of enough quality food for an active and healthy life. This might include home food production, transportation, and exchange systems for food. This dimension is also concerned that food is available in socially acceptable ways. “Access” is achieved when the resources required to acquire food are met and can include economic or physical resources. “Utilization” refers to the intake of sufficient and safe food and the physical, social and human resources to transform food into meals. The final dimension, “Stability”, recognizes that food insecurity can be transitory, cyclical or chronic [4]. All these dimensions are necessary for food insecurity to be understood as a continuum that progresses from uncertainty and anxiety about access to sufficient and appropriate food at the household level, to the extreme condition of hunger among children because they do not have enough to eat [5]. Food insecurity is caused by a range of circumstances, including low or unstable employment, poor food supply, illness, and financial pressures; people often fall prey to food deprivation not so much because food is unavailable on the market, but because their access to food is constrained [6].

Household food insecurity and nutritional vulnerability have significant health implications for both adults and children, with food insecurity having the potential to exacerbate existing health inequalities [7]. Food insecure households have been shown to consume low cost, poor quality foods, high in energy, fat, and sugar, and low in nutritional value [8]. Among adults, food insecurity is associated with an increased risk of chronic conditions including diabetes and hypertension [9], and anxiety, depression, and mood disorders [10]. Among children, food insecurity is associated with poor general health [11], atypical or problematic behaviour, and delayed development [12]. Severe food insecurity can also lead to nutritional deficiencies [13], and weight loss or weight gain [14]. Food insecurity may be transient, in that people move in and out of food insecurity as their circumstances change, though increasingly people are experiencing chronic food insecurity [15,16], and as a result, are turning in greater numbers to charities that provide food relief [17,18,19]. Given the very serious potential implications of food insecurity, understanding the ways in which researchers investigate food insecurity, and the findings of such research, is important for the creation of policy and practice responses.

Food security research in Australia is relatively new, with much of the research conducted in the past decade. The infancy of this work, and the variety of approaches taken to measure food security in general [20], means that researchers in Australia, like those investigating food insecurity internationally, have adopted a number of approaches to the measurement of food insecurity. Globally, many researchers rely on the United States Department of Agriculture (USDA) Household Food Security Survey Module (HFSSM) when measuring household food insecurity. The HFSSM consists of a set of questions based on the overall experience of household food insecurity, administered through a survey; results can be reported as a continuous score of severity, or with cut-off points through which households are classified into four categories [21]. The HFSSM is a household-level self-report of uncertain, insufficient or inadequate food access, availability, and utilization that can assesses the food security situation of adults and children within a household, but not the food security status of individuals in the household. The HFSSM contains 18 questions about the food security situation in the household over the previous 12 months. The measure can also be shortened to a 10-item and 6-item sub-scale that allow for the differentiation between low levels of food security and very low levels of food security. One limitation of this scale is that it does not capture reasons beyond financial constraints, poor resources, and compromised eating patterns and consumption. Webb et al. [6] suggest that the HFSSM is a valid way to measure household food insecurity in the USA, and with some modification the HFSSM has been used more widely in the Americas [22,23,24]. Other household food security scales, which use context-specific questions that differ from the HFSSM, have been used in developing countries based on in-depth assessments and understanding of the local experiences with food insecurity [25,26]. For example, in rural Bangladesh, themes within the scale included meals, cooking, and specific ingredients used in cooking and food management strategies [25], while in Burkina Faso, themes included agricultural production and decisions about production and uses of food, cooking and eating patterns, perception of food quality, coping and strategies [26].

Based on the HFSSM is the Food Insecurity Experience Scale (FIES), created by the Food and Agriculture Organization (FAO) [27,28]. The FIES consists of eight questions and measures the access dimension of food security, particularly financial aspects, but also the availability of quality food. Unlike the HFSSM, the FIES can measure individual food insecurity. The result of the FIES is a score of food insecurity severity. This instrument is relatively new; however, it has been used to assess food insecurity in a number of different countries [29,30,31].

An alternative to the HFSSM is the single-item measure often incorporated into population level health surveys. The single item askes: “In the last 12 months was there any time you have run out of food and not been able to purchase more?” This question has been included in the Australian National Health Survey (NHS), conducted every three years as an indicator for the severity of food insecurity. The most recent national level food security data collected by the Federal Government identifies the prevalence of food insecurity in the general Australian population to be approximately 5% [32]. This figure is said to be around 3% in Victoria, New South Wales (NSW), and South Australia, closer to 5% in Queensland, Western Australia, and the Northern Territory, and around 6% in Tasmania [33]. Researchers have investigated the validity of this question in determining food insecurity and have found that most likely results in under reporting food insecurity [7,34,35]. This single item originates from the Radimer/Cornell Food Security Scale (a measure of food insecurity) [36]. The Radimer/Cornell Food Security Scale was developed in the 1990s through in-depth interviews with women with children living at home who had experienced hunger [37]. These interviews resulted in two conceptualizations of hunger that were then used to create a food security scale: one that referred to insufficient food intake and going without food and the second that encompassed problems with household food supply, quality of diets, feelings about the situation, and coping.

Given the large amount of literature and research that has investigated food security over the past four decades, it is perhaps unsurprising that there also exists a number of systematic reviews on the topic. For example, there are systematic reviews that investigate the relationship between food insecurity and mental health [38], the role of food banks in alleviating food insecurity [17], and the experiences of students and food insecurity [39]. More closely related to this research are two recent reviews that have investigated measurement tools. The first is that of Ashby et al. [20], who investigated the use of multi-item tools in measuring food insecurity and explored which of the four dimensions of food security these tools measure. This research identified eight tools, each of which assessed the “access” dimension of food security, with two partially assessing the “food utilization” and “stability over time” dimensions. This study concludes with the suggestion that a tool should be created that measures all four dimensions of food insecurity. The second is that of Marques et al. [40] who sought to identify and characterize experience-based household food security scales. This research found that while there are a number of instruments available, most have been developed in the USA, and have undergone limited testing for reliability and validity.

This current systematic review is interested specifically in the food security research in Australia. While having many similarities with the USA, the food landscape and response to food insecurity from both government and non-government actors differs in Australia. While the HFSSM and the single item are the most common methods of identifying and classifying food insecurity, researchers and those in the food aid sector also employ other methods such as using measures of food consumed or food knowledge as a proxy for food security or attendance at a food bank as an indicator for food insecurity [19,41]. This lack of coherency concerning how food insecurity is measured likely has an impact on the reported prevalence, which likely influences policy and practice responses. This review seeks to (1) systematically investigate the peer reviewed literature that purports to investigate food insecurity in Australia, (2) identify the breadth of research being conducted in Australia, including the instruments used and the populations under study, and (3) provide an overview of the severity of food insecurity in Australia as presented by these studies.

## 2. Methods

A systematic search was undertaken to identify all food security research conducted in Australia. Key search terms were “food insecurity” OR “food security” OR “food availability” OR “food utilisation” OR “food access” AND “Australia”. Searched databases included EBSCOhost (including Academic Search Complete, CINAHL Complete, Global Health, and MEDLINE), and SCOPUS. In order to gain a full collection of articles that reported on food security research in Australia, no limits were placed on publication dates. Only peer reviewed articles published in English were considered; unpublished articles, books, theses, dissertations, and non-peer reviewed articles were excluded.

**Figure 1:**
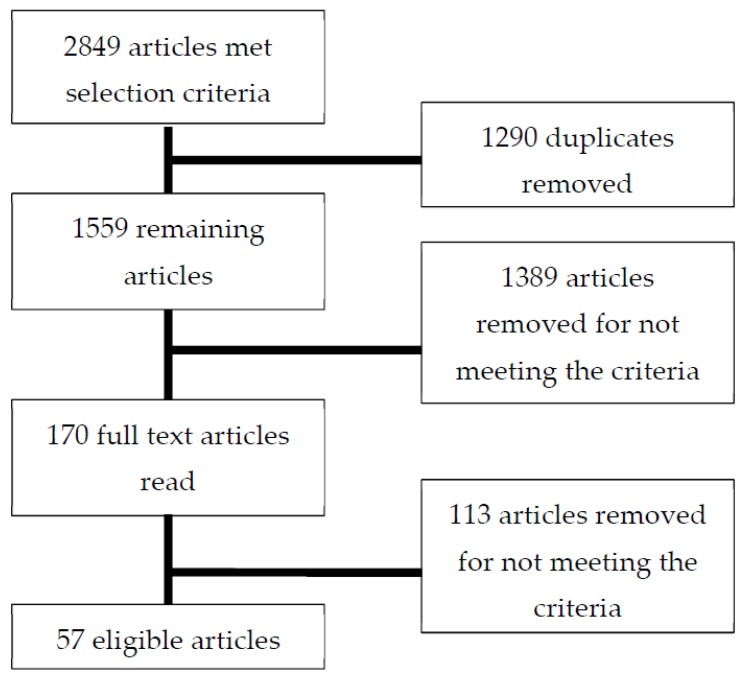
Flow chart of articles meeting search criteria, number of articles excluded, and final number of articles meeting inclusion criteria for review.

Two authors (FHM and BCH) reviewed all articles to identify relevant studies. Articles underwent a three-step process (see Figure 1). All articles were downloaded into EndNote X7, duplicates were identified and removed. Articles were first screened by title and abstract based on the inclusion and exclusion criteria above. Any article that clearly did not meet the inclusion criteria was removed at this stage, any that did, or possibly could meet the inclusion criteria on further inspection, were retained. Full text of the remaining articles were obtained for further assessment. Two authors (FHM and BCH) independently read all 170 articles that remained at this stage and decided whether each article had met the inclusion criteria. Any articles at this stage that clearly did not meet the inclusion criteria were removed. Of those that were retained, disagreements were discussed and settled by consensus. The reference lists of articles were also read to identify any further studies that met inclusion criteria—this did not result in any additional articles.

Articles that discussed a program in response to food insecurity but did not measure food insecurity (for example References [42,43]) were excluded, as were other systematic reviews (for example References [17,20,44,45]) and narrative reviews [46]. As we were interested in all studies that purported to measure food insecurity in Australia, studies that discussed food insecurity, as either the standard measured construct or as a construct created by the authors but termed food insecurity, were included. While the food aid sector in Australia reports on food insecurity, (for example References [19,47]), these reports generally do not include a complete description of the method used to collect data and often use food bank attendance as a proxy for national food insecurity level; these reports have therefore been excluded from this review.

Data were extracted from each article by two authors (FHM and BCH). Data were extracted into a spreadsheet that allowed for the capture of specific information across all included articles. Data extracted at this stage included: State; Location; Population group; Findings; Testing an intervention (Y/N); Primary method; Measured food security (Y/N); Method for determining food insecurity; Prevalence of food insecurity; Participant numbers; and Participant description.

## 3. Results

The search identified 2849 articles, of which 1290 were duplicates. The titles and abstracts of the remaining 1559 articles were read, with 1389 articles excluded as they did not refer, either directly or indirectly, to food insecurity research in Australia, leaving 170 articles for further investigation. The full text of the 170 articles were reviewed; of these, 17 articles were excluded as they were identified as review articles, and 97 articles were excluded as they did not meet the inclusion criteria on closer inspection. The remaining 57 studies have been included in this review (see Table 1).

### 3.1. General Characteristics

**Figure 2:**
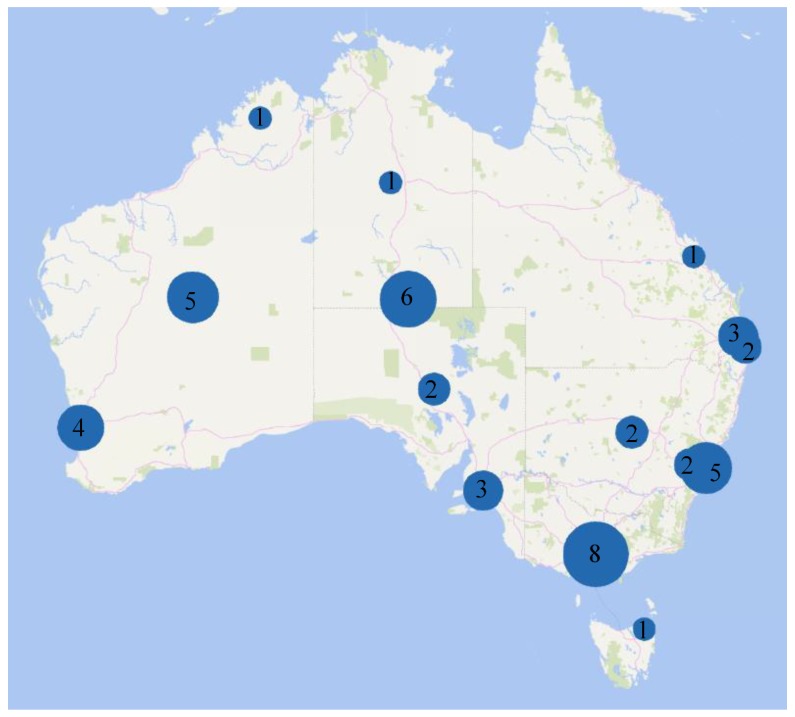
Geographic distribution of 57 studies included in a systematic review of the measurement of food insecurity in Australia.

Participant numbers in studies reviewed ranged in size from the smallest study with only six participants [48] to population level studies with over 57,000 participants [32]. Most food insecurity research was conducted in the state of Victoria, where 18 studies were conducted, followed by NSW and Western Australia, with nine studies each (see Figure 2).

Studies employed a range of methods. Two thirds of studies (*n* = 38) employed a quantitative methodology, utilizing a survey or audit [1,2,7,16,34,49,50,51,52,53,54,55,56,57,58,59,60,61,62,63,64,65,66,67,68,69,70,71,72,73,74,75,76,77,78,79,80,81], while the remainder (*n* = 18) were qualitative. Thirteen studies used interviews [41,67,82,83,84,85,86,87,88,89,90,91], four studies employed focus groups [48,92,93,94], and one utilized photo voice [95].

### 3.2. Food Insecurity Measurement

**Figure 3:**
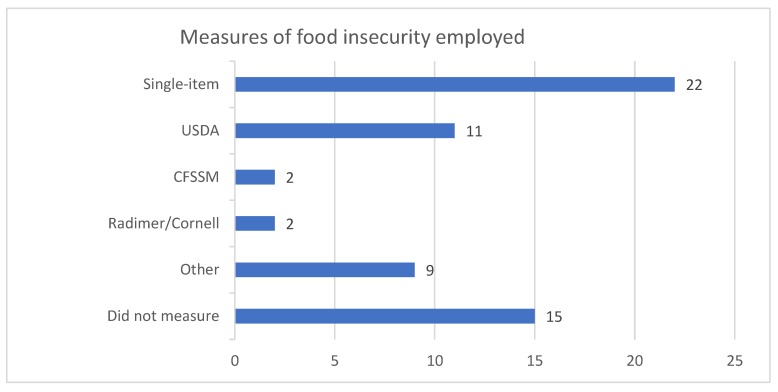
Tools used to measure food insecurity in the studies included in this systematic review.

Thirty-three studies directly measured food insecurity (see Figure 3). The most common way to measure food insecurity was via the single item: “In the last 12 months, were there any times that you ran out of food and couldn’t afford to buy more?” This question has been criticized by many researchers for regularly under-reporting food insecurity in population-based studies [7,34]. This question (or a slight variation) was included in 22 studies [1,7,32,50,51,52,54,56,58,61,62,63,64,66,70,71,72,73,77,79,80,92]. Food insecurity measured by the single item ranged from 2.0%, reported in a study of older Australians [52], to 76%, reported in a study of food availability in remote Aboriginal communities [58].

Eleven studies included some form of the HFSSM. For example, Allen and Wilson [54] used four questions from the HFSSM, Hughes et al. [61] utilized the 8-item tool (excluding the questions relating to child hunger) as well as the single item question as above, as did Crawford et al. [92] who, in addition to the single item and the HFSSM, also included a question relating to finding food in other places, such as friends or drop-in meal programs. Gichunge et al. [60], Ramsey et al. [16], Kleve et al. [33], and Gallegos, Ramsey, and Ong [59] used the full 18-item tool, while Nolan et al. [7], and Ramsey et al. [16] describe using a 16-item scale (that is the 18-item scale without the two frequency questions). Kleve et al. [33] also included the new Household Food and Nutrition Security Survey (HFNSS). Both McKay and Dunn [2] and Micevski, Thornton, and Brockington [65] used the 6-item USDA tool, plus a follow-up question around hunger. Food insecurity measured by the USDA tool (or variants) ranged from 18% in a population of resettled African refugees in Queensland reported in a study by Gichunge et al. [60], to over 90% in a population of asylum seekers using a food bank in Victoria reported in a study by McKay and Dunn [2].

The Radimer/Cornell instrument was used in two articles [68,69]. These two articles report on the same dataset, where 13% of the population were identified as food insecure.

Three studies compared food insecurity status between the USDA HFSSM and the single item [7,61,92]. Crawford et al. [92] used a nine-item version of the USDA HFSSM with a 30-day reference period, finding 70% of young people experiencing homelessness were food insecure, compared to 58% of the same population who were experiencing food insecurity when measured by the single item question with a 12-month reference period. Hughes et al. [61] compared food insecurity prevalence against the 8-item HFSSM and the single item, with results indicating 46% and 12.7%, respectively. Nolan et al. [7] employed the 18-item USDA HFSSM and the single item question to measure food insecurity among disadvantage populations, finding a higher prevalence with the HFSSM than the single item, 21.9% and 15.8%, respectively. Allen and Wilson [54] used a modified version of the USDA HFSSM including just four items in addition to the single item question; however, given only four questions were included from the USDA HFSSM, a score for food insecurity could not be calculated from this scale, with the prevalence reported for each individual score. Kleve et al. [34] compared the USDA HFSSM with the new Household Food and Nutrition Security Survey (HFNSS), finding the HFNSS reporting higher food insecurity compared to the HFSSM, 57% and 29%, respectively, providing further evidence that the USDA HFSSM may be underreporting food insecurity in Australia.

In addition, ten studies included a proxy measure of food insecurity, but were unable to describe prevalence. For example, in a study investigating the lived experiences of food insecurity among Aboriginal people with disabilities, Spurway and Soldatic [85] asked questions about alternative food access, including fishing and crabbing, with the assumption that all Aboriginal people were food insecure and were using these alternatives as a way to mitigate this insecurity. Wicks, Trevena, and Quine [83] asked urban soup kitchen customers questions about severity and frequency of hunger. Almost 80% described going an entire day without food, and this result was used as a proxy for food insecurity. Burns et al. [56] measured two indicators (i.e., financial and physical restriction) of food insecurity, finding no evidence between financial food insecurity and the purchase of fruit and vegetables, or nutritionally recommended foods, though there was some evidence to suggest that financial and physical restrictions were associated with more frequent purchasing of chain-brand fast food. Cuesta-Briand, Saggers, and McManus [94] investigated access to food through focus groups and interviews for those living with type 2 diabetes. Edmond et al. [74] provided an indication of food security by asking participants about the foods they had consumed in the past 24 h and if they had sought any information about food security. Godrich et al. [91] and Godrich et al. [81] investigated determinants of food insecurity through surveys and interviews with caregivers as a way to determine if children were receiving sufficient nutrition. Friel et al. [51] used the indicative question from the Household Income and Labour Dynamics in Australia (HILDA) Survey: ‘Since the beginning of this year, did you go without meals because of a shortage of money?’ to identify the relationship between food insecurity and drought, finding that among people living in drought-affected areas, those who consumed more discretionary food items were more likely to experience distress. Lindberg, Lawrence, and Caraher [41] asked users of food aid about their experiences of going without food, budgeting, and their use of food charities as a way to understand food insecurity, finding that charitable food services are an important part of the food security safety net. Finally, Pollard et al. [67] investigated issues related to supply chains and food retailers to gain an understanding of observed or assumed food insecurity, finding that supply-chain issues result in increased food costs, and as a result, increased food insecurity in remote populations.

Two studies reported on food security outcomes but did not report on data collection measures. Crawford et al. [57] reported on structural barriers to achieving food security by homeless young people in Australia; however, there was no mention of the measurement of food insecurity or reporting of food insecurity status for this group within the article. Myers et al. [96] reported that 13% of supported playgroup families were food insecure compared to only 5% of mainstream playgroup families; however, there was no mention of how this information was collected in the article.

### 3.3. Investigation of Food Insecurity in Different Population Groups

The articles reviewed focused on a range of populations. Eight studies measured food insecurity in the general Australian public [7,32,34,50,51,54,56,77]. These studies were typically a secondary analysis of national or state-based population level surveys. Six of these studies used the single item question to indicate food insecurity. Two studies that reported on the single item from Australia wide data reported different rates of food insecurity, with Friel et al. [51] using the Household, Income and Labour Dynamics in Australia (HILDA) data from 2007 to report food insecurity of 1.6% and Temple [71] using the National Health Survey of 2005 to report food insecurity at 5.1%. Two studies investigated food insecurity in Victoria; Burns et al. [56] used the single item question from the VicLANES data which sampled from metropolitan Melbourne finding food insecurity at 8.1%, while Kleve et al. [32] used the single item question from the Victorian Population Health Survey (2006–2009) finding food insecurity between 4.9%–5.5%. Using the single item question from their respective state-based population health surveys, Foley et al. [50] found food insecurity at 7.0% in South Australia, while Lê et al. [77] found food insecurity at 5% in north eastern Tasmania. Using the USDA HFSSM for their population level studies, Allen and Wilson [54] did not report a single score as they used a truncated scale, while Kleve et al. [34] reported food insecurity of 29% based on the USDA HFSSM and 57% using the HFNSS. Seven studies reported on Aboriginal and Torres Strait Islanders [58,63,64,67,73,74,95]; these studies investigated the food insecurity situation for both urban and rural Aboriginal and Torres Strait Islanders. Four studies measured food insecurity using the single item [58,63,64,73], with food insecurity ranging from 76% in a remote area of the Northern Territory, to 20.3% in Victoria. The remaining three studies investigating food insecurity among Aboriginal and Torres Strait Islanders did not include a measure of food insecurity, instead investigating experiences of food [95], alternative access to foods [85], and the experience of receiving advice about food insecurity [74].

Ten studies reported on children or young people [49,53,55,57,76,82,86,90,92,93]; however, only three of these studies measured and reported on food insecurity with findings ranging from 70% food insecure [92] to 20.1% food insecure [76]. Three studies reported on university students [59,61,65], all finding higher rates of food insecurity among students than the general population. Five reported on older people [52,68,69,70,79], with food insecurity ranging from 2% to 13%. Refugees and asylum seekers were the focus of three studies [1,2,60] where food insecurity ranged from 18% to 91%, this large range could be ascribed to the ability of asylum seekers and refugees on different visas to access financial support. Finally, two articles focused on those accessing food banks [40,83], neither of these studies measured food insecurity directly, however, both reported that users of food banks employed a range of strategies to mitigate hunger.

### 3.4. Interventions to Mitigate Food Insecurity

Of the 56 articles reviewed, only five reported on an intervention that was aimed at limiting or reducing food insecurity [48,55,82,87,90]; an additional four studies reported on research collected within an intervention but did not report on the intervention itself [58,62,76,93]. One study reported on the evaluation of a social café meals program aimed at reducing food insecurity and improving social inclusion [87], with findings suggesting that participating in the program increased access to food, with the café setting identified as important in promoting community cohesion. Another reported on the evaluation of a Food Cent$ pilot program [48], a program designed to illustrate the financial benefits of healthy eating. This evaluation found that Food Cent$ could potentially increase knowledge about nutrition, however, the small sample size (*n* = 6) is a limitation making it difficult to identify causal links. Two studies reported on FoodMate, a program focused on improving food literacy as a way to improve food security. An evaluation of the pilot returned largely inconclusive results, attributed to high attrition rates with the authors highlighting the difficulties in including people at risk in long-term programs [55]. The second paper to evaluate this program included past FoodMate program graduates and found that the increased nutrition knowledge gained through the program could lead to increased food security; however, this study was also small, with only 10 participants [90]. Finally, a study investigating the role of a community meals program operating for Aboriginal people in Victoria through interviews with 23 staff, found that such programs may offer access to safe, affordable, nutritious food that is also culturally and socially acceptable; however, sustainability of the program was identified as an ongoing problem [82].

## 4. Discussion

This review has investigated the breadth of food insecurity research in Australia, finding that over the past 15 years researchers have focused on a range of populations including Aboriginal and Torres Strait Islanders, refugees and asylum seekers, older and younger populations, university students, and those receiving charitable food assistance. Researchers have used a range of tools to measure food insecurity, from the validated and reliable USDA HFSSM, to the less reliable single item measure. Of concern to the authors were the number of studies that purported to measure food insecurity, but used a proxy measure, a non-valid measure or failed to mention the data collection methods in sufficient detail, if at all. Given the already limited amount of information relating to food insecurity in Australia, this lack of research rigor is of great concern.

Various single-item and multi-item tools have been developed to determine the prevalence of food insecurity at a population level. As shown in this review, the measurement of food insecurity in Australia is commonly limited to a single item asking whether anyone in a household has run out of food in the preceding 12 months and has been unable to purchase more due to a lack of money. This review found food insecurity as identified by this question ranged from 2.0% in older Australians [52] to 76% in remote Aboriginal communities [58]. Australian studies suggest that the single-item measure underestimates the prevalence of food insecurity by at least 5% [7], compared with more comprehensive multi-item measures [34,57,61]. The use of this single item in the Australian Health Survey has consistently reported a national level of food insecurity of approximately 5%; due to this low prevalence, food insecurity data are not routinely collected in Australia—in the past decade, food security has only been measured twice; in 2004–2005 and 2011–2012.

Given the limitations of the single-item food security measurement tool, the more sensitive, multi-item tool developed by the USDA is commonly used to estimate food insecurity prevalence and severity [7,20]. The 18-item USDA HFSSM can determine severity and prevalence of food insecurity and was identified as being employed in eleven studies included in this review. This tool takes into consideration the multi-dimensional nature of food insecurity, typically within a 12-month reference period; however, it is also valid for a 6-month or 30-day reference period, and exhibits good reliability (based on a Cronbach’s alpha greater than 0.70) [97].

Given the different methods for data collection, including the wide range of tools used to measure food insecurity, and the different population groups targeted, it is difficult to compare the reported food insecurity across the 57 studies included in this review. However, in general, this review identified a higher prevalence of food insecurity in studies employing the HFSSM—from 18% [60], to over 90% [2], compared with those using the single item—with findings of food insecurity from 2.0%, [52], to 76% [58], with the highest food insecurity reported in populations of people seeking asylum [2] and remote Aboriginal populations [58].

While the HFSSM has been validated in the USA, where it has been shown to provide accurate measures of food insecurity with the capability to distinguish between varying degrees of food insecurity, it has a number of limitations for use in Australia [98]. This tool focuses on a single dimension of food insecurity (affordability), and not the other three, equally important dimensions, which limits its applicability to the broader Australian context of food insecurity, where access, utilization, and sustainability are key considerations of food insecurity. In addition to affordability, food security in Australia is influenced by the presence of food deserts [99], challenges with transport to food stores [56], regionality and availability of foods in remote areas [100], the nature of the retail environment [101], the welfare and employment system [8], and the availability of culturally appropriate foods [2], all things that need to be taken into consideration when reporting on food insecurity at a population level. The HFSSM is also unable to identify food security at the individual level, rather measuring food insecurity at the household level, potentially hiding individual differences in severity of experienced food insecurity among members of a household. The 12-month reference period also limits the ability of the instrument in capturing severe but short-term hunger [35]. As such, there remains a gap for a novel tool that can measure the experience of food insecurity in the Australian context.

Several limitations within the studies reviewed have been identified. A number of studies had very small sample sizes, making it very difficult to generalize results [41,48,55,85,86,87,88,89,95]. Poor response rates were reported by several studies; for example, Gallegos, Ramsey, and Ong [59] only received a response rate of 6.7%, while Barbour et al. [55] reported more than half of participants were lost on follow-up. Other studies had methodological problems, for example as acknowledged by Allen et al. [87], interviews in their study were brief and conducted by inexperienced researchers, with data saturation not reached. Finally, it should be noted that there have been no longitudinal studies conducted in Australia to investigate the long-term impacts or experiences of food insecurity.

### Limitations

There are some limitations of this review that should also be acknowledged. While every attempt was made to ensure this review was comprehensive, additional articles may have been missed. Given that this is the first review of its kind, with the inclusion of several databases and broad key terms, the authors are confident that there is little information that is not presented here. Given the variety of approaches taken to measure food insecurity, there are challenges in comparing the outcomes of different studies. However, as this is not a meta-analysis, the authors do not feel this should invalidate the findings.

## 5. Conclusions

This review is the first of its kind to investigate the breath and scope of food security research conducted in Australia. This research found that researchers are using a variety of methods to collect information about food insecurity, including a single item question that has been found to return an inaccurate measure of food insecurity. As a result of the variety of methods employed, there is little understanding of the true prevalence and severity of food insecurity in Australia. Based on the findings of this review, the authors suggest more work is needed to create a measure of food insecurity that will suit Australia, which will allow researchers to gain a clear understanding of the prevalence of food insecurity in the Australian community.

## Figures and Tables

**Table 1:** Summary of 57 studies included in systematic review.

Ref.	Location	Population Group	Study Aim	Findings	Testing an Intervention?	Primary Method	Measured Food Security	Method for Determining Food Insecurity	Prevalence of Food Insecurity	Participants
[95]	Geelong, Victoria	Aboriginal (urban)	To work with an urban aboriginal community to understand meaning of food and food insecurity.	Participants were concerned about hungry children and were filling up to satiate hunger with high energy foods. Vegetable content was low, and some people found cooking a socially isolating and boring experience. Food was associated with family harmony (keeping children happy through food). Family recipes were viewed with pride.	No	Photo voice	No	N/A	N/A	10; mostly female aged 20–30
[54]	Australia	General public and university students	To examine if materialists have an elevated concern about food availability.	Those who can be described as materialists are not experiencing food insecurity. Materialists have food stored at home and tend to be obese.	No	Survey	Yes	Modified USDA—4 items, 12-month reference, single item	24% “food bought didn’t last, no money for more”, 16% “couldn’t afford balanced meals”, 17% “cut or skipped meals”, 12% “ate less than felt I should”	334; general public (210) and university students (124).
[87]	Melbourne, Victoria	Socially isolated and food insecure	To explore the ability of a café meals program to address social exclusion and food insecurity.	An evaluation of a social meals program found that participants had improved access to food, with the setting of the café identified as important in promoting community cohesion.	Yes (Social Café Meals Program)	Interviews	No	N/A	N/A	18; Café owners/workers and program members.
[55]	Melbourne, Victoria	Young people at risk	To access the impact of the FoodMate program by SecondBite in at risk young people on their dietary intake and quality, cooking confidence and food independence.	Difficult to get people at risk to be involved in a long-term program. Some positives around improved nutrition. Confidence in basic meal preparation returned to baseline at 4–6-week follow-up, some improvements to confidence in the ability to “buy, store, prepare and enjoy nutritious food at all times from non-emergency sources” over the course of the intervention (high attrition may negate useful findings).	Yes (FoodMate)	Survey	No	N/A	N/A	9; Young people, median age 20 (half homeless, half experiencing food insecurity at program start).
[46]	Western Australia	Mothers with poor mental health	To investigate if the Food$ents program influenced behaviours and attitudes toward food and food selection and perpetration.	Food$ents might be a good way to increase knowledge about nutrition.	Yes (Food$ents)	Focus groups and supermarket receipts (pre and post program)	No	N/A	N/A	6; female who had experienced poor mental health and had children under 5.
[96]	Adelaide, South Australia	Young people experiencing homelessness	To determine the food sources and acquisition practices used by homeless youth in Adelaide.	Homeless young people use a range of sources to procure food including theft, visiting welfare agencies, begging, and deliberate incarceration.	No	Survey and interviews	No	N/A	N/A	150; 15–24 years, 54% male.
[86]	Adelaide, South Australia	Young people experiencing homelessness	To report on street life and the extent to which homeless youth justify their behaviours.	Homeless young people exhibit prosocial behaviours of sharing food with other people, begging together, and protecting each other. Moral stance influenced how young people sourced food.	No	Interviews	No	N/A	N/A	15; 15–23 years, 9 female and 6 male.
[72]	Melbourne, Victoria	Main food shopper in each house	To describe the associations between demographic and individual and area level Socioeconomic variables and household access to food due to a lack of money, ability to lift and transport foods.	Difficulty lifting groceries is a factor in food insecurity for low-income people and those with no access to a car. The elderly and those born overseas were more likely to report difficulty lifting groceries. Single individuals with or without children were more likely to report having no money for food. Those experiencing disadvantage were 12 times more likely to report having no money for food.	No	Survey	Yes	Single Item	8.10%	2564; stratified population to include a gradient of socio-economic status.
[56]	Melbourne, Victoria	General public	To examine the associations between financial, physical and transport conditions that may restrict food access and the purchase of foods.	No evidence found between financial food insecurity and the purchase of fruit and veg, or nutritionally recommended foods. No evidence that difficulty lifting is associated with purchasing healthy foods.	No	Survey	Yes	Single item	8.1%	2564
[92]	Sydney, New South Wales	Young people experiencing homelessness	To investigate issues associated with food insecurity and nutrition in young people experiencing or at risk of homelessness.	Young people living independently had higher food insecurity than young people living in supported accommodation services. Participants reported skipping meals and low consumption of fruit and vegetables.	No	Survey	Yes	9-item USDA: 30-day reference, CFSSM, and single item measure: 12-month reference.	70% (USDA), 58% (single item), CFSSM not reported	50; 14–26 years, 29 female
[57]	Sydney, New South Wales	Young people experiencing homelessness	To examine the extent of food insecurity and the eating patterns of young people accessing support from a homeless service.	Participants described daily experiences of food shortages and hunger and associated anxiety. More severe food insecurity is said to be experienced by the more disadvantaged participants; however, food insecurity not measured.	No	Focus groups	No	N/A	N/A	48; 15–24 years, 29 female, 18 male, 1 transgender
[94]	Perth, Western Australia	Low income, diabetes patients, half Aboriginal	To explore food security issues faced by low-income earners living with type 2 diabetes to explore the effect of socio-economic disadvantage.	Participants were aware of what consisted of a “healthy diet” was but were not always able to attain it. Indigenous participants compared to non-Indigenous participants were more likely to rely on others for supply of foods. The perceived high cost of diabetes-appropriate foods was problematic for participants. Social networks helped low-income participants to access food.	No	Interviews and focus groups and	Proxy	Access to food	Not reported	38; mostly female, aged over 65.
[73]	Australia	Aboriginal	To examine the prevalence and patterning of psychological distress among Aboriginal Australians adults and compare these with corresponding non- Aboriginal data.	Running out of food was associated with very high psychological stress.	No	Survey	Yes	Single item	24.6%	5417; 18–64 years
[74]	Australia (all states except Victoria and Tasmania)	Aboriginal	To assess delivery of social and emotional wellbeing services to the families of Aboriginal children.	Audits of data collected during visits to primary health care centres suggests child food insecurity is an issue for Indigenous communities (11%), however rates of follow-up for these children is low (30%).	No	Client audit	Proxy	Advice about food security	10.60%	2466 children aged between 3 and 59 months. Mostly from remote areas
[75]	Perth, Western Australia	Community gardeners and coordinators	To report on the attitudes of community gardeners toward local food, and how these attitudes fit into the context of Alternative Food Networks as a response to food security.	Community gardens can be used to produce food to address food insecurity at the same time they can be useful in education about the food system.	No	Interviews	No	N/A	N/A	35
[58]	Northern Territory	Aboriginal (very remote)	To explore the availability, variety, and frequency of consumption of traditional foods and their role in alleviating food insecurity in remote Aboriginal Australia.	Traditional foods are an important component of the diet of Aboriginal people.	No—conducting research in an intervention context, SHOP@RIC, did not report on intervention.	Survey	Yes	Single item	76%	73 Aboriginal primary household shoppers, 97% female
[50]	South Australia	General public	To estimate the extent of food insecurity in South Australia and its relationship with a variety of socio-economic variables.	Those with lower education, the unemployed (only significant in bivariate analysis, not multi) lower incomes, people in households that were unable to save, Aboriginal households, and households with three or more children were more likely to be food insecure.	No	Survey	Yes	Single item	7%	19,037
[51]	Australia	General public	To investigate the associations between food insecurity and drought and mental health.	Psychological stress is associated with food insecurity. No relationship between drought exposure and food insecurity.	No	Survey	Yes	Single item	1.6%	5012
[1]	Perth, Western Australia	Refugees	To identify food insecurity and examine its association with socio-demographic factors in a group of newly arrived refugees.	Refugees experience food insecurity for a variety of reasons related to income. Many described feelings of shame in accessing food aid.	No	Survey	Yes	Single item	71%	51 refugees in Australia for less than 12 months, over 18 years
[59]	Brisbane, Queensland	University students	To investigate the food insecurity status of university students.	Food insecurity more common in those living out of home and on low income. Food insecure less likely to have adequate diets, more likely to report fair/poor health, more likely to report deferral of studies.	No	Survey	Yes	18-item USDA, 12-month reference	25.5%	810; mostly young females
[60]	South East Queensland	Refugees	To assess the interaction of food insecurity, social support, and vegetable intake among refugees.	Higher than population level food insecurity, but lower than other studies of refugees. Those with low education and no social support more likely to experience food insecurity. No difference in vegetable intake between food secure and insecure.	No	Survey	Yes	18-item USDA, 6-month reference	18%	383 participants from 71 households. Many children (67%), mostly female (88.7%)
[91]	Western Australia	Key informants	To determine whether there is a relationship between food security determinants and adequate vegetable consumption among children in regional and remote Western Australia.	Food insecurity is influenced by inequalities in availability, price, promotion and quality of healthy food.	No	Interviews	Proxy	Food situation	Not reported	20 mostly female from regional areas
[76]	Western Australia	School children and their care givers	To ascertain the prevalence of food insecurity among regional and remote Western Australian children and to determine which socio-demographic factors predicted food insecurity.	Receipt of government benefit and relative disadvantage are predictors of child food insecurity.	No—conducting research in an intervention context, but did not report on intervention.	Survey	Yes	CFSSM	20.1%	438 mostly female children and female care givers.
[81]	Western Australia	Care givers	To explore how determinants of food security affect children in regional and remote Western Australia across food availability, access and utilization.	Determinants that predicted vegetable consumption included food availability, promotion and access to foods.	No	Survey	Proxy	Food security determinants	Not reported	187 mostly female, with a medium age of 41 years.
[93]	Adelaide, South Australia	Children, low SES	To explore how children negotiate food practices in community environments that were the target of a public health obesity and healthy lifestyle initiative.	Food insecurity suggested present. Hunger and obesity were stigmatized.	No—conducting research in an intervention context but did not report on intervention.	Observation and focus group	No	N/A	N/A	Unspecified; Children 10–14 years, involved with charitable food relief organizations
[61]	Gold Coast, Queensland	University students	To identify and describe prevalence, distribution and severity of food insecurity, and related behavioural adaptions, among a sample of Australian university students.	Food insecurity significantly associated with renting, boarding, or sharing accommodation. Students on low incomes and government assistance more likely to be food insecure. Those who reported food insecurity more likely to report overall lower health status.	No	Survey	Yes	8-item USDA (current year at university) and single item	46% (USDA),12.7% (singe item)	399; representative of student body
[32]	Victoria	General population	To investigate the prevalence and frequency of food insecurity in low- to middle-income households over time and identify factors associated with food insecurity.	Low- and middle-income households more likely to experience food insecurity. Inability to get help from friends, and dependent children were strongly correlated with FI.	No	Survey	Yes	Single item	4.9–5.5%	57,056
[34]	Victoria	General public	To investigate the psychometric properties, validity and reliability of a newly developed measure of food insecurity.	The HFNSS reported higher food insecurity than the USDA	No	Survey	Yes	18-item USDA and HFNSS	29% USDA; 57% HFNSS	134 mostly female, aged 26–45
[89]	South Australia	Low-income single parents	To apply a livelihoods framework approach as an analytical lens and organizational structure to explore strategies single parents use to maintain food security.	Food acquisition is constrained by finances.	No	Interviews	No	N/A	N/A	8 low-income single parents
[77]	Dorset, Tasmania	General public	To investigate the impact of socio-economic factors on food security and the coping strategies used when food shortages occur.	Food choice was influenced by availability, supply, and access (particularly cost of transport to shops). Most participants felt nothing could be done to improve their physical and financial access to healthy foods.	No	Survey and focus groups	Yes	Single item + how often have you run out of nutritious food?	5% (single item), ran out of nutritious food 10.5% weekly or fortnightly, 15.8% during the previous 12 months	364 (survey) 45 (focus group participants); mostly female, more than half over 55
[62]	Sydney, New South Wales	First-time mothers	To assess dietary behaviours during pregnancy among first time mothers, and to investigate the relationship between these behaviours and demographic characteristics.	Low levels of fruit and vegetable consumption and low levels of food insecurity. Mothers in households with lower incomes were more likely to consume fewer vegetables. High levels of fast food and soft drink intake were reported.	No—conducting research in an intervention context but did not report on intervention.	Survey	Yes	Single item	5%	409 first time mothers at 26–36 weeks. Average age 26 years
[41]	Melbourne, Victoria	Previous or current users of food charities	To understand food aid users’ experience of food insecurity and gain evidence for effective responses.	Alternatives to “cap in hand” food aid and more respectful services needed. Users of services need to be included in any solutions.	No	Interviews	Proxy	Going without, budgeting, use of food charities	N/A	12 users of emergency food aid
[63]	Victoria	Aboriginal	To explain the relationship between food insecurity and Aboriginal and Torres Strait Islanders.	Aboriginal and Torres Strait Islanders more likely to experience food insecurity. Food insecurity in this study can be explained by age, household income, smoking, obesity and ability to get help from friends.	No	Survey	Yes	Single item	20.3%	339; 51.4% male, over 18 years
[64]	Victoria	Aboriginal	To identify determinants of health for Aboriginal adults compared to non-Aboriginal adults.	Authors state that food insecurity is a psychosocial risk factor, Aboriginal and Torres Strait Islanders experience food insecurity at a higher rate.	No	Survey	Yes	Single item	20.3%	339
[2]	Melbourne, Victoria	Asylum seekers	To explore the food insecurity status of asylum seekers.	High rates of food insecurity in asylum seekers, related to lack of income and lack of cooking facilities.	No	Survey	Yes	7-item USDA—30-day reference	91%	56 food bank users
[78]	Perth, Western Australia	Food outlet owners	To investigate issues relating to food insecurity.	Food outlets did not provide a range of healthy food choices.	No	Survey	No	N/A	N/A	99
[90]	Melbourne, Victoria	Young people experiencing homelessness	To investigate the impact of Secondbites’s FoodMate program for young people experiencing homelessness.	The program may have positive impacts of dietary behaviours both immediately after the program and at the 2-year mark. Homeless young people may misunderstand what is meant by food security (classifying themselves as food secure, when still accessing food relief).	Yes (FoodMate)	Interviews and focus groups	No	N/A	N/A	11
[65]	Melbourne, Victoria	University students	To assess the prevalence of food insecurity in university students.	More likely to be food insecure if living out of home.	No	Survey	Yes	7-item USDA—current university year	48%	124 university students
[66]	New South Wales	People with psychosis	To examine the association of social dysfunction with food security status, fruit intake, vegetable intake, meal frequency and breakfast consumption in people with psychosis.	Rates of social dysfunction, significant food insecurity, and intakes of fruits and vegetables below recommendations in people with psychosis.	No	Survey	Yes	Single item	25.30%	221; 60% male
[82]	Victoria	Community food program staff	To explore the role of community food programs operating for Aboriginal people and their perceived influence on food access and nutrition.	Community food programs may offer access to safe, affordable, nutritious, culturally and socially acceptable food.	Yes (Community Food Program)	Interviews	No	N/A	N/A	23 staff from a range of community food programs. 20 women, 3 men. Majority Aboriginal.
[96]	Victoria	Play group parents	To compare nutrition and active play of children aged 0–4 years attending Supported Playgroups and mainstream services and to compare access, understanding and application of health information within these families.	Supported play groups (those for disadvantaged families) demonstrated more vulnerability, with families experiencing difficulties accessing, understanding and applying positive health advice.	No	Survey	Yes	Unspecified	13% (Supported play group), 5% (mainstream play group).	412 parents from mainstream, and supported play groups.
[7]	Sydney, New South Wales	General public (in socially disadvantaged areas)	To determine the prevalence of food insecurity within an urban population of social disadvantage in readiness for a local health promotion response.	Three food insecurity coping strategies identified: cutting variety of food, delaying bill payment, carer skipping meals or eating less. Renting, capacity to save, health status and having children within the household were strongly associated with food insecurity.	No	Survey	Yes	16-item USDA single item tool—past 12 months	15.8% (single item), 21.9% (USDA)	1719; 76% male, 54% not completed high school
[67]	Western Australia	Store managers in Aboriginal communities	To explored remote community store managers’ views on issues related to improving food security to inform health policy.	Freight costs and irregular deliveries contribute to high prices and limited range of foods. Store managers described a practice where community members would deposit money with the store manager to ensure money for store goods at a later date.	No	Interviews	Proxy	Perceptions of customer food insecurity	63% said no FI, 52% said hunger because people did not have enough money to buy food.	33
[52]	New South Wales	Older	To identify the extent of food insecurity amongst older Australians, and the characteristics of those who experience this condition.	Those experiencing food insecurity have poorer health, limited financial resources, non-home ownership, are more likely to live alone, and to need assistance at home. As females aged, they reported lower food insecurity.	No	Survey	Yes	Single item	2%	8881 aged over 65 years and living independently
[79]	Melton, Victoria	Older	To investigate the experiences and barriers to food security of community-dwelling older people.	The single item question may under report food insecurity in older Australians. Although low incidence of food insecurity, many reported previous use of a food bank and a range of other indicators of food insecurity. Social networks were an important mechanism of acquiring culturally appropriate food.	No	Survey and focus groups	Yes	Single item	3%	37 mostly female, between 58–85 years, mostly on a pension (83%)
[53]	Brisbane, Queensland	Children	To investigate associations between food insecurity, sociodemographic and health factors and dietary intakes among adults residing in disadvantaged urban areas.	Children with a parent born outside of Australia were less likely to experience food insecurity. Children in food insecure households were more likely to miss days at school and were more likely to have emotional and/or behavioural problems.	No	Survey	Yes	16-item USDA	34%	185; aged 25–45
[16]	Brisbane, Queensland	Low SES	To investigate associations between food insecurity, sociodemographic, and health factors and dietary intake among adults residing in disadvantaged areas.	Food insecurity was associated with lower income households, poor mental health, poor general health, and increase hospital visits.	No	Survey	Yes	18-item USDA	25%	505; half female, mostly aged 30–50.
[68]	Blue Mountains, New South Wales	Older	To estimate the prevalence of food insecurity and to identify associated characteristics in a cohort of older Australians.	High rate of food insecurity in older Australians. Women and younger respondents (less than 70) were more likely to be FI. Those living on a welfare payment, living alone, and renting were more likely to be food insecure. Being a current smoker was also a strong predictor.	No	Survey	Yes	Radimer/Cornell	13%	3068; over 45 years.
[69]	Blue Mountains, New South Wales	Older	To examine the relationships of food security and diet quality with health-related quality of life in a cohort of older Australians.	Food insecure respondents had poorer quality of health. Those with poor food insecurity were more likely to have poor mental and physical health.	No	Survey	Yes	Radimer/Cornell	Not included (reported elsewhere)	2642; over 45 years
[84]	Victoria	Government	To analyse inter-governmental partnership approaches facilitating local government’s response to food insecurity.	Good government partnerships can build the capacity of local government to act on food security initiatives and help to legitimize food security work within local governments. Local government staffing arrangements were a limiting factor.	No	Interviews	No	N/A	N/A	27 government staff and program evaluators.
[88]	Rockhampton, Queensland	Supply chain actors governing food security	To consider the ways that different actors within the community mobilized resources, information and relationships to ensure food security for the city during the flooding crisis of 2011.	Poor formal decision making and communication among supply chain actors in time of crisis can lead to food insecure communities.	No	Interviews and secondary data analysis	No	N/A	N/A	13 government, community groups, industry, emergency services
[85]	Kimberley region, West Australia	Aboriginal (remote)	To investigate the impact of chronic food insecurity on the daily lives of remote Aboriginal Australians.	Participants use alternative methods to obtain food when food insecure. Poor access to transport, economic insecurity, and inadequate government social assistance compounded food insecurity. Social support networks were important to obtain traditional foods.	No	Interviews	Proxy	Alternative food access (fishing and crabbing)	N/A	16 people with disabilities and Aboriginal family members who were carers
[70]	Australia	Older	To examine the prevalence of food insecurity among older persons, the characteristics of the food insecure and the association between food insecurity and well-being.	People living alone are more likely to be food insecure, as are those on a low income. Food insecurity were more likely to report poorer health and quality of life.	No	Survey	Yes	Single item	2.80%	4650; over 55 years
[71]	Australia	General public	To examine the prevalence and correlates of the severity of food insecurity, and to uncover potential health and nutrition outcomes.	Food insecurity is a result of financial constraint and insufficient access to food. Those who are food insecure also have poor health.	No	Survey	Yes	Single item	5.1%	19,501
[80]	Victoria	Low-income women	To investigate the associations between sociodemographic factors and both diet indicators and food security among socio-economically disadvantaged populations in two different (national) contextual settings.	Food insecurity was more likely in unmarried, unemployed, and low-income women.	No	Survey	Yes	Single item	14.70%	1340, mostly aged 18–45
[83]	Sydney, New South Wales	Soup kitchen users	To describe the experiences of food insecurity among participants who participated in interviews at a charity-run soup kitchen in urban Sydney, Australia.	People who attend soup kitchens are reliant on these charities for a large proportion of food. These participants had good dietary knowledge, and so did not require cooking classes or the like but were hungry because of low income and lack of cooking facilities.	No	Interviews	Proxy	Frequency and severity of hunger	Over half reported eating less than 3 meals per day, most reported meal skipping.	22; mostly single middle-aged and male.
